# Supplementary material for: Individual retrotransposon integrants are differentially controlled by KZFP/KAP1-dependent histone methylation, DNA methylation and TET-mediated hydroxymethylation in naïve embryonic stem cells
Source: Epigenetics Chromatin. 2018 Feb 26;11:7. doi: 10.1186/s13072-018-0177-1 (PMC6389204; doi:10.1186/s13072-018-0177-1)
Supplement: Supplementary file 11 — Additional file 11. Pattern analysis. [file 13072_2018_177_MOESM11_ESM.zip › Patterns analysis/DataTables/extensions/Scroller/examples/large_js_source.html]

Scroller example - Client-side data source (50,000 rows)


# Scroller example Client-side data source (50,000 rows)

This example is completely artificial in that the data generated is created on the client-side by
just looping around a Javascript array and then passing that to DataTables. However, it does show quite
nicely that DataTables and Scroller can cope with large amounts of data on the client-side quite
nicely. Typically data such as this would be Ajax sourced and server-side processing should be
considered.

Please be aware that the performance of this page will depend on your browser as the array of data
is generated - for example IE6 will crawl!

| ID | First name | Last name | ZIP / Post code | Country |
| --- | --- | --- | --- | --- |

- Javascript
- HTML
- CSS
- Ajax
- Server-side script

The Javascript shown below is used to initialise the table shown in this
example:

`$(document).ready(function() {
var data = [];
for ( var i=0 ; i<50000 ; i++ ) {
data.push( [ i, i, i, i, i ] );
}
var oTable = $('#example').dataTable( {
data: data,
deferRender: true,
dom: "frtiS",
scrollY: 200,
scrollCollapse: true
} );
} );`

In addition to the above code, the following Javascript library files are loaded for use in this
example:

- ../../../media/js/jquery.js
- ../../../media/js/jquery.dataTables.js
- ../js/dataTables.scroller.js

The HTML shown below is the raw HTML table element, before it has been enhanced by
DataTables:

This example uses a little bit of additional CSS beyond what is loaded from the library
files (below), in order to correctly display the table. The additional CSS used is shown
below:

The following CSS library files are loaded for use in this example to provide the styling of the
table:

- ../../../media/css/jquery.dataTables.css
- ../css/dataTables.scroller.css

This table loads data by Ajax. The latest data that has been loaded is shown below. This data
will update automatically as any additional data is loaded.

The script used to perform the server-side processing for this table is shown below. Please note
that this is just an example script using PHP. Server-side processing scripts can be written in any
language, using the protocol described in the
DataTables documentation.

## Other examples

### Examples

- Basic initialisation
- State saving
- Client-side data source (50,000
  rows)
- Server-side processing (5,000,000
  rows)
- API

Please refer to the DataTables documentation for full
information about its API properties and methods.  
Additionally, there are a wide range of extras and
plug-ins which extend the capabilities of
DataTables.

DataTables designed and created by SpryMedia Ltd © 2007-2014  
DataTables is licensed under the MIT license.
